# Transcriptome analysis of nitrogen-starvation-responsive genes in rice

**DOI:** 10.1186/s12870-015-0425-5

**Published:** 2015-02-03

**Authors:** Wenzhu Yang, Jinmi Yoon, Heebak Choi, Yunliu Fan, Rumei Chen, Gynheung An

**Affiliations:** Department of Plant Molecular Systems Biotechnology and Crop Biotech Institute, Kyung Hee University, Yongin, 446-701 Korea; Department of Crop Genomics and Genetic Improvement, Biotechnology Research Institute, Chinese Academy of Agricultural Sciences, Beijing, 100081 China; National Key Facility for Crop Gene Resources and Genetic Improvement (NFCRI), Biotechnology Research Institute, Chinese Academy of Agricultural Sciences, Beijing, 100081 China

**Keywords:** N-starvation, *Oryza sativa*, Transcription factors, Transcriptome sequencing

## Abstract

**Background:**

Nitrogen (N), a critical macronutrient for plant growth and development, is a major limiting factor in most agricultural systems. Microarray analyses have been conducted to investigate genome-wide gene expression in response to changes in N concentrations. Although RNA-Seq analysis can provide a more precise determination of transcript levels, it has not previously been employed to investigate the expression of N-starvation-induced genes.

**Results:**

We constructed cDNA libraries from leaf sheaths and roots of rice plants grown under N-deficient or -sufficient conditions for 12 h. Sequencing the libraries resulted in identification of 33,782 annotated genes. A comparison of abundances revealed 1,650 transcripts that were differentially expressed (fold-change ≥ 2) due to an N-deficiency. Among them, 1,158 were differentially expressed in the leaf sheaths (548 up-regulated and 610 down-regulated) and 492 in the roots (276 up, 216 down). Among the 36 deficiency-induced genes first identified via RNA-Seq analyses, 34 were subsequently confirmed by qRT-PCR. Our RNA-Seq data identified 8,509 multi-exonic genes with 19,628 alternative splicing events. However, we saw no significant difference in alternative splicing between N-sufficient and -deficient conditions. We found 2,986 novel transcripts, of which 192 were regulated under the N-deficiency.

**Conclusion:**

We identified 1,650 genes that were differentially expressed after 12 h of N-starvation. Responses by those genes to a limited supply of N were confirmed by RT-PCR and GUS assays. Our results provide valuable information about N-starvation-responsive genes and will be useful when investigating the signal transduction pathway of N-utilization.

**Electronic supplementary material:**

The online version of this article (doi:10.1186/s12870-015-0425-5) contains supplementary material, which is available to authorized users.

## Background

The macronutrient nitrogen (N) is an essential component of numerous important compounds, including amino acids, proteins, nucleic acids, chlorophyll, and some plant hormones. This element is a major limiting factor in most agricultural systems. Because the N-utilization efficiency strongly influences crop productivity, a vast amount of N fertilizers is applied to maximize yields. However, it is estimated that more than half of that N is lost from the plant–soil system, with unused N fertilizers severely polluting the environment [1]. Thus, N-uptake efficiency must be increased to improve productivity and reduce pollution.

During periods of N-starvation, various deficiency-responsive genes function to support plant survival by increasing the level of chlorophyll synthesis [2], altering root architecture [3], improving N-assimilation [4], enhancing lignin content [5], and changing the amounts of sugars and sugar phosphates [6]. Nitrate transporter genes (*NRT*s) are responsible for the high-affinity NO_3_^−^ transport system and stimulate lateral root growth. *Arabidopsis NRT2.1* plays a major role in NO_3_^−^ uptake and determines root architecture by controlling lateral root formation [7]. The ammonia transporter gene *AtAmt1.1*, which is highly expressed in the roots, also restructures this architecture under limited-N conditions [8]. The plant-specific Dof1 transcription factor (TF) from maize also functions to increase N-assimilation [9]. In Dof1-overexpressing *Arabidopsis* plants, genes are up-regulated under N-starvation to encode enzymes for carbon skeleton production [9]. Those transgenic plants also show markedly elevated amino acid contents, reduced levels of glucose, and improved growth during periods of N-deficient stress [9]. Overexpression of *glutaminesynthetase1* in tobacco and maize is associated with significant gains in plant heights, dry weights, and kernel numbers [10,11]. Overexpression of *NADH*-*glutamatesynthase* in rice and *alanine aminotransferase* in canola and rice also causes increases in grain weights [12] and biomass [13,14]. An early nodulin gene, *OsENOD93-1*, responds to both increases and reductions in N supplies. Furthermore, transgenic rice plants over-expressing *OsENOD93-1* have greater shoot dry biomass and seed yields [15].

Microarray analyses have been conducted to investigate genome-wide gene expression in response to changes in N conditions. Wang *et al.* [16] studied gene responses in *Arabidopsis* plants that were first grown for 10 d with ammonium as the sole N source, then treated with 250 mM nitrate for 20 min. That analysis identified 1,176 nitrate-responsive genes in the roots and 183 in the shoots. Peng *et al.* [17] monitored expression profiles from *Arabidopsis* plants grown under nitrate-limiting or -sufficient conditions. There, N-starvation altered transcript levels for 629 genes, with 340 being up-regulated and 289 down-regulated. Palenchar *et al.* [18] identified over 300 genes regulated by interactions between carbon and N signaling in *Arabidopsis*. Bi *et al.* [19] detected differential expression of genes under mild or severe chronic N stress. Plant responses were much more pronounced under severe conditions.

With ‘Minghui 63’ rice, Lian *et al.* [20] applied EST microarrays to examine expression profiles under low-N stress. In seedling roots, 473 responsive genes were identified, with 115 being up-regulated and 358 down-regulated. Beatty *et al.* [21] generated transgenic rice plants that overexpress alanine aminotransferase. Comparisons of transcriptomes between the transgenic plants and controls revealed that 0.11% and 0.07% of those genes were differentially regulated in the roots and shoots, respectively. Cai *et al.* [22] analyzed the dynamics of the rice transcriptome at 1 h, 24 h, and 7 d after N-starvation treatment. In all, 3,518 genes were identified, with most being transiently responsive to such stress.

Xu *et al.* [23] performed a genome-wide investigation to detect miRNAs that responded to either chronic or transient nitrate-limiting conditions in maize. They identified miRNAs showing overlapping or unique responses as well as those that were tissue-specific. Humbert *et al.* [24] reported that the concomitant presence of N and a water deficit affected expression much more than was anticipated in maize. This research group also revealed how the interaction between those two stresses shaped patterns of expression at different levels of water stress as well as during the recovery period. Finally, Brouillette and Donovan [25] identified five genes that had markedly different responses to nitrogen limitations in *Helianthus anomalus* when compared with *H. petiolaris* and *H. annuus*.

Although microarray analyses have been extensively used for the past few decades, RNA-Seq analysis can more precisely measure transcript levels and allow for the absolute quantification of gene expression. However, RNA-Seq has not previously been employed to investigate N-deficiency-induced genes. Here, we report transcriptome profiles for 1,650 N-starvation-responsive genes from rice for which expression was altered in the roots or shoots due to an N-limitation.

## Results and discussion

### RNA-Seq analysis of N-deficiency stress-responsive genes

Through microarray analyses, early-responsive genes have been detected in rice roots but not in leaves when sampled after 20 min, 1 h, and 2 h of N-starvation [20,22]. Cai *et al*. have monitored such genes after long-term (1- and 7-d) treatments with limited-N [22]*.*

**Figure 1:**
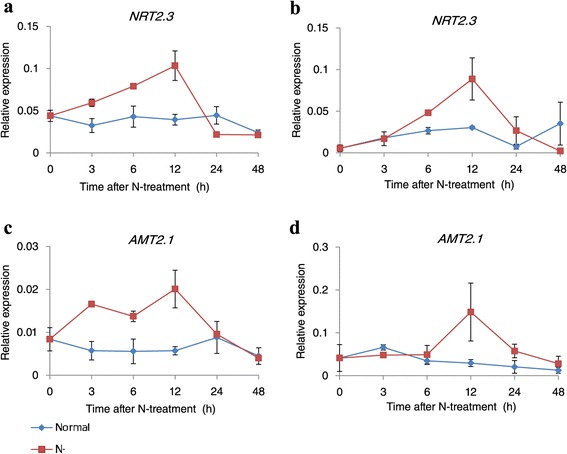
**Induction kinetics of N-starvation-induced genes.** Leaf sheaths **(a and c)** and roots **(b and d)** of rice seedlings at six-leaf stage were harvested at 3 h, 6 h, 12 h, 1 d, and 2 d after N-starvation and -sufficient treatments were applied. *NRT2.3* **(a and b)** and *AMT2.1* **(c and d)** were used to investigate induction kinetics.

**Figure 2:**
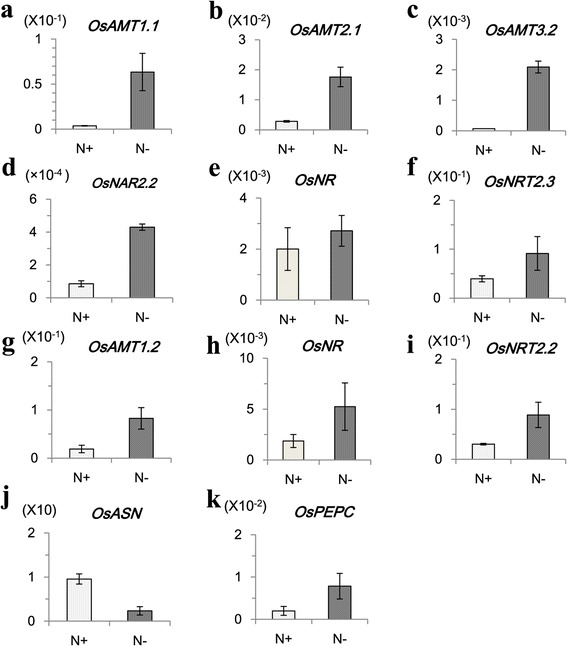
**Analyses of N-metabolism genes by RT-PCR. (a-f)** Transcript levels of *OsAMT1.1*, *OsAMT2.1*, *OsAMT3.2*, *OsNAR2.2*, *OsNR*, and *OsNRT2.3* were measured in leaf sheaths sampled from seedlings grown under N-sufficient (N+) or -deficient (N-) conditions. **(g-k)** Transcript levels of *OsAMT2.1*, *OsNR*, *OsNRT2.2*, *OsASN*, and *OsPEPC* were measured in roots sampled from seedlings grown under N+ or N- conditions. Levels were relative amounts against *OsUbi* expression.

To identify additional responsive genes, we transferred rice seedlings at the six-leaf stage to an N-deficient hydroponic solution. Leaf sheaths and roots were harvested after 3 h, 6 h, 12 h, 1 d, and 2 d. We used two previously identified N-starvation-induced genes -- *NRT2.3* and *AMT2.1* -- to investigate induction kinetics (Figure 1). In both sheaths and roots, transcript levels were increased upon starvation, peaking at 12 h before declining to basal levels after 1 d. This trend was consistent with earlier reports [2,3,26]. Therefore, we selected the 12-h point for RNA-Seq analyses to distinguish between our results and those of studies that had investigated only very early- or late-responsive genes. Because expression of stress-responsive genes is mostly transient, we believed our data would be valuable for finding a new class of N-starvation-responsive genes. Leaf sheaths and roots were harvested from plants grown under deficient or sufficient conditions. RT-PCR analyses were used to determine the response of several N-metabolism genes, including *OsAMT1.1*, *OsAMT1.2*, *OsAMT2.1*, *OsAMT3.2*, *OsNAR2.2*, *OsNR*, *OsNRT2.2*, *OsNRT2.3*, *OsPEPC*, and *OsASN*. Significant changes in expression were revealed in the 12-h N-deficient samples (Figure 2).

We constructed eight cDNA libraries from two biological replicates of leaf sheaths and roots from plants grown under deficient or sufficient conditions. Sequencing those libraries resulted in the identification of 40,756,549 and 41,703,971 paired-end reads (202-nucleotide read length) from the sheaths and roots, respectively. The generated reads were then aligned to the rice genome (IRGSP/RAP build 5 data set) [27,28] by applying Bowtie [29] and TopHat2 programs [30]. In all, 86% of the reads from the sheaths and 69% from the roots were mapped to the reference genome, for which nearly 87% were correctly aligned and approximately 98% of them had unique locations in that genome (Table 1).

**Table 1:** **Analysis of RNA-Seq data from rice seedlings**

**Category**	**Leaf sheaths**	**Roots**
Total reads	40,756,549	41,703,971
Mapped reads^a^	34,863,681 (85.5%)	28,612,070 (68.6%)
Paired-end mapped reads^b^	29,808,452 (85.2%)	25,178,621 (88.1)
Uniquely mapped reads^c^	33,964,261 (97.4%)	28,057,411 (98.1%)

^a^Reads were aligned to the rice genome by Bowtie and TopHat2.

^b^Paired-end mapped reads.

^c^Reads were aligned to only one location in the genome.

Transcript profiles of the RNA-Seq data were analyzed by calculating the reads per kilo base per million reads (RPKM). The sequenced RNA covered 33,782 annotated genes, accounting for 86.2% and 86.7% of those genes in the sheaths and roots, respectively. In addition, 2,986 novel transcripts were detected. Transcripts with low RPKM values were removed because they may not have been reliable due to low abundance or statistical faults. Among the 36,768 transcripts, 26,699 had RPKM ≥ 2. Of those, 22,992 were present in the leaf sheaths, 24,087 in the roots, and 18,319 in both. We identified 6,319 transcripts that were uniquely expressed in the sheaths (2,612) or roots (3,707).

Among the transcriptionally active transcripts, the top 500 most highly expressed were identified from the leaf sheath (Additional file 1) and roots (Additional file 2) under N-limited conditions. In both organ types, the most frequent transcripts functioned for protein synthesis, protein degradation, photosynthesis, stress responses, TFs, and DNA synthesis. Transcripts involved in lipid metabolism, transport, secondary metabolism, and amino acid metabolism were also common.

### Differential expression of transcripts due to N-deficiency

Comparing transcript abundances revealed 1,650 transcripts that were differentially expressed (fold-change ≥ 2; *p* ≤ 0.05) due to a deficient N supply (Additional files 3 and 4). Among them, 1,158 were differentially expressed in the leaf sheaths and 492 in the roots. Of those identified in the N-deficient sheaths, 548 transcripts were up-regulated and 610 transcripts were down-regulated. In the N-deficient roots, 276 transcripts were up-regulated and 216 were down-regulated. To gain insight into the effect of N status on transcript expression profiles, we illustrated expression patterns with a heat map obtained via hierarchical cluster analysis (Additional file 5). This clustering revealed the relatedness of the various transcripts.

Transcription factors are important for controlling the expression of other genes. Several TFs have been described in plants exposed to limited N. For example, an R2R3-type MYB TF, CmMYB1, is a central regulator of N-assimilation in *Cyanidioschyzon merolae* and enhances the expression of *CmNRT*, *CmNAR*, *CmNIR*, *CmAMT*, and *CmGS* under N-starvation [4]. A member of the *Arabidopsis* GATA TF gene family, At5g56860, is inducible by nitrate; loss-of-function mutants cause reduced chlorophyll levels and downregulation of the genes involved in carbon metabolism [2]. In *Arabidopsis*, *ANR1* encodes a MADS box protein and is induced by nitrate. When expression of this gene is suppressed, lateral root proliferation is altered due to a reduction in sensitivity to NO_3_^−^ [3]. Of the 1,650 transcripts that we found differentially expressed under an N-deficiency, 86 were identified as TFs, covering 28 families (Table 2; Additional file 6). This included one TF each from the GATA, Dof, and MADS families. The AP2/EREBP and WRKY TF families are the two largest families responsive to this deficiency. Here, six AP2/EREBP TF members were increased in the sheaths and seven in the roots under stress. Twelve WRKY members were induced in the sheaths versus none in the roots. It will be valuable in future investigations to determine whether these TFs also play a critical role in the N-starvation response and plant development.

**Table 2:** **TFs differentially expressed in roots and leaf sheaths due to N-deficiency**

**TF Family**	**Leaf sheaths**		**Roots**	
	**Up**	**Down**	**Up**	**Down**
ABI3/VP1		1		
AP2/EREBP	6	2	7	1
ARF		1	1	
AS2(LOB)			3	
bHLH	1	4		
bZIP	1			
C2H2	4	3	1	
C2C2-CO-like			1	
C2C2-GATA		1		
CPP		1		
DBB (Orphans)	1			
Dof		1		
E2F/DP		1		
FAR1		1		
GARP-G2-like		2	1	
GRAS		1		
GRF		2		
HB	2	1		
HSF	2	1		
LSD			1	
MADS	1			
MYB	3	1	3	
NAC	3		1	2
SBP		1		
TCP		1		
Trihelix			1	
WRKY	12			
ZF-HD	1			
Total	37	26	20	3

**Figure 3:**
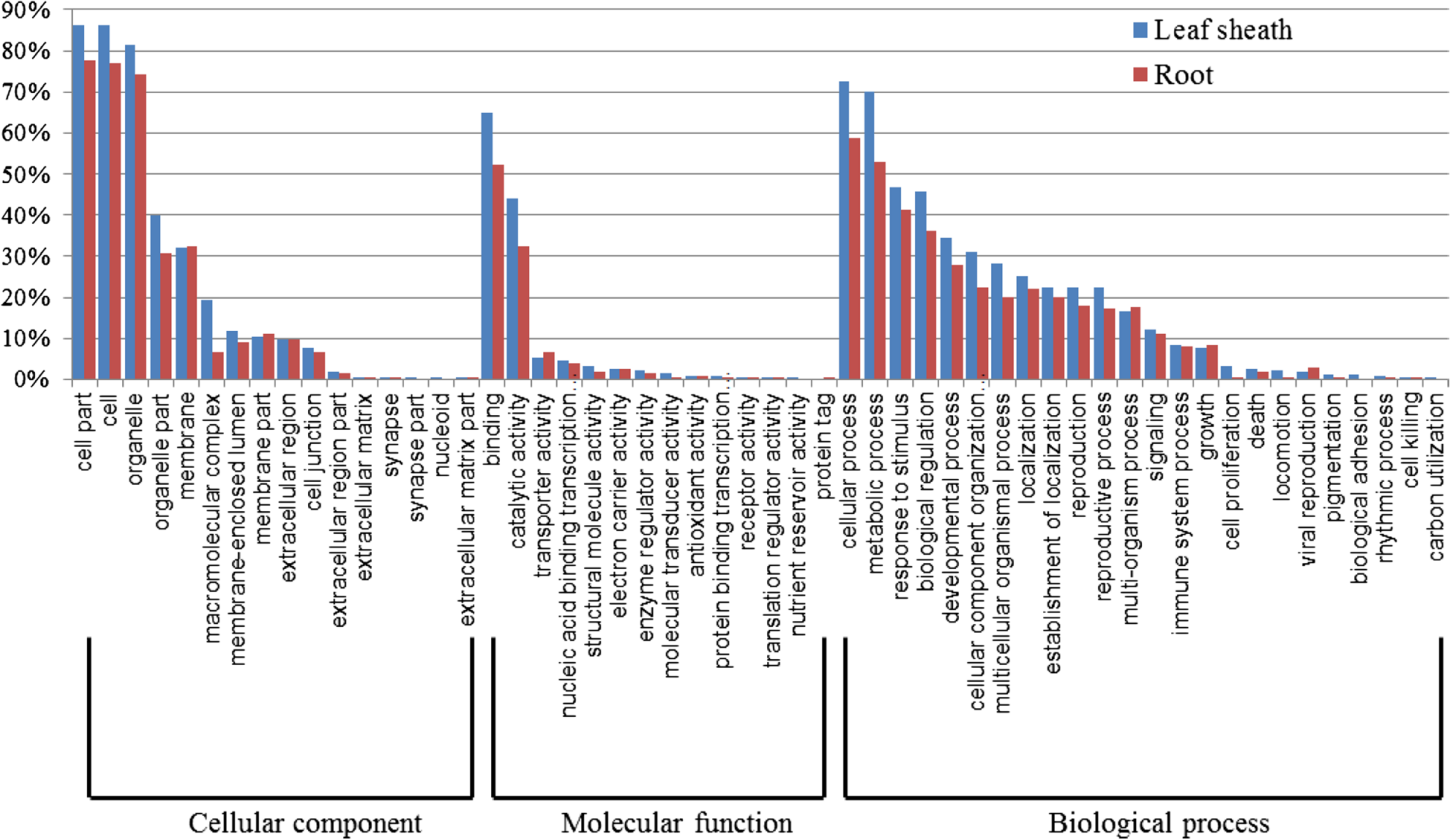
**GO annotation clusters of differentially expressed genes.** Gene Ontology functional enrichment analysis of differentially expressed genes in leaf sheaths and roots. Based on sequence homology, 1,650 genes were distributed among 3 main categories: Cellular Component (16 functional groups, dominated by ‘cell part’), Molecular Function (14 groups, dominated by ‘binding’), and Biological Process (24 groups, dominated by ‘cellular process’).

We classified the 1,650 differentially expressed genes into 54 functional groups by GO analysis (Figure 3). The dominant terms were ‘cell part’ (GO:0044464) in Cellular Component, ‘binding’ (GO:0005488) in Molecular Function, and ‘cellular process’ (GO:0009987) in Biological Process. In the third category, more than 30% of the genes for ‘metabolic process’ (GO:0008152), ‘response to stimulus’ (GO:0050896), and ‘biological regulation’ (GO:0065007) responded to N-starvation. ‘Cellular process’ accounted for 72.4% and 58.3% of the starvation-related genes in the leaf sheath and root, respectively. ‘Metabolic process’ genes made up 70.0% and 53% in the sheath and root, respectively; while those proportions were 46.7% (sheath) and 41.1% (root) for ‘response to stimulus’ and 45.7% (sheath) and 36.3% (root) for ‘biological regulation’.

### Confirmation by real-time PCR

Our RNA-Seq data appeared to be quite reliable for genes up-regulated by N-starvation, with 34 of the 36 deficiency-responsive genes first identified via RNA-Seq analyses subsequently being confirmed by qRT-PCR (Table 3). Only two could not be verified in that latter examination. By contrast, the identification of down-regulated genes by RNA-Seq was less reliable. Among 12 examined, eight were later confirmed through qRT-PCR (Table 4).

**Table 3:** **RNA-Seq results from leaf sheaths confirmed by real-time PCR**

**Locus ID**	**RNA-Seq log**_**2**_ **(N-/N+)**	**RT-PCR log**_**2**_ **(N-/N+)**	**Putative function**
LOC_Os03g32230	4.35	2.25	Zinc finger protein
LOC_Os04g52090	2.32	1.56	Ethylene-responsive transcription factor 4
LOC_Os03g62200	2.26	2.66	Ammonium transporter protein
LOC_Os04g40410	2.25	4.89	High affinity nitrate transporter
LOC_Os02g08440	2.20	2.23	WRKY71
LOC_Os02g43790	1.89	1.27	Ethylene-responsive transcription factor 1A
LOC_Os03g55540	1.72	2.66	ZOS3-18 - C2H2 zinc finger protein
LOC_Os03g60560	1.67	1.08	Zinc finger protein ZAT12
LOC_Os03g60080	1.58	2.68	NAC domain-containing protein 67
LOC_Os01g50820	1.48	1.21	Transporter, major facilitator family
LOC_Os12g02440	1.47	1.89	WRKY transcription factor 46
LOC_Os12g07640	1.47	1.97	Myb-related protein
LOC_Os11g03540	1.46	1.63	Ethylene-responsive transcription factor
LOC_Os05g03900	1.43	1.85	WRKY109
LOC_Os06g41100	1.32	2.32	bZip, transcription factor
LOC_Os07g12340	1.32	1.38	NAC domain-containing protein 67
LOC_Os02g43330	1.30	1.43	Homeobox-associated leucine zipper
LOC_Os02g53130	1.26	2.12	Nitrate reductase
LOC_Os01g53220	1.26	−1.40	Heat stress transcription
LOC_Os02g43170	1.20	3.19	B-box zinc finger family protein
LOC_Os04g50770	1.20	2.21	MYB-related protein Zm1
LOC_Os04g45810	1.13	2.45	Homeobox-leucine zipper protein HOX22
LOC_Os02g49840	1.08	3.32	MADS-box transcription factor 57
LOC_Os01g54600	1.07	2.87	WRKY13
LOC_Os09g28354	1.07	1.07	Heat stress transcription factor B-1
LOC_Os04g43070	1.02	1.72	Ammonium transporter protein
LOC_Os03g42200	−2.00	−1.02	Dof zinc finger domain containing protein
LOC_Os03g08620	−1.60	−2.38	B3 DNA binding domain-containing protein
LOC_Os04g44440	−1.59	0.88	TCP family transcription factor
LOC_Os01g74540	−1.56	1.00	GATA zinc finger domain-containing protein
LOC_Os05g34110	−1.42	0.97	Homeodomain-related
LOC_Os10g42490	−1.26	−2.03	Homeobox and START domain-containing proteins
LOC_Os02g39140	−1.06	1.01	Helix-loop-helix DNA-binding domain containing protein
LOC_Os06g43220	−1.04	−2.12	AP2 domain-containing protein

Expression ratios for RNA-Seq data were calculated with the DESeq program. All ratios are presented as Log_2_N-deficient/N-sufficient. Negative values indicate that expression was reduced under N-starvation.

**Table 4:** **RNA-Seq results from roots confirmed by real-time PCR**

**Locus ID**	**RNA-Seq log**_**2**_ **(N-/N+)**	**RT-PCR log**_**2**_ **(N-/N+)**	**Putative function**
LOC_Os02g02190	3.51	1.55	Transporter, major facilitator family
LOC_Os02g53130	2.57	2.97	Nitrate reductase
LOC_Os06g10780	2.01	3.76	Ethylene-responsive transcription factor ERF014
LOC_Os06g01480	1.66	1.91	NAC domain-containing protein 7
LOC_Os01g50720	1.41	2.23	MYB-related protein Hv33
LOC_Os02g57490	1.41	2.07	LOB domain-containing protein 16
LOC_Os02g41580	1.14	1.97	Phosphoenolpyruvate carboxylase kinase
LOC_Os10g07510	1.08	0.67	LOB domain-containing protein 18
LOC_Os03g32230	1.05	1.76	Zinc finger protein 1
LOC_Os02g40710	1.04	2.12	Ammonium transporter protein
LOC_Os01g64790	−4.97	−2.78	Ethylene-responsive transcription factor ERF110
LOC_Os03g18130	−1.73	−2.02	Asparagine synthetase
LOC_Os08g10080	−1.53	−1.01	NAC domain-containing protein 21/22
LOC_Os11g08210	−1.06	−1.84	NAC domain-containing protein 71

Expression ratios for RNA-Seq data were calculated with the DESeq program. All ratios are presented as Log_2_N-deficient/N-sufficient. Negative values indicate that expression was reduced under N-starvation.

### Validation by GUS assays

**Figure 4:**
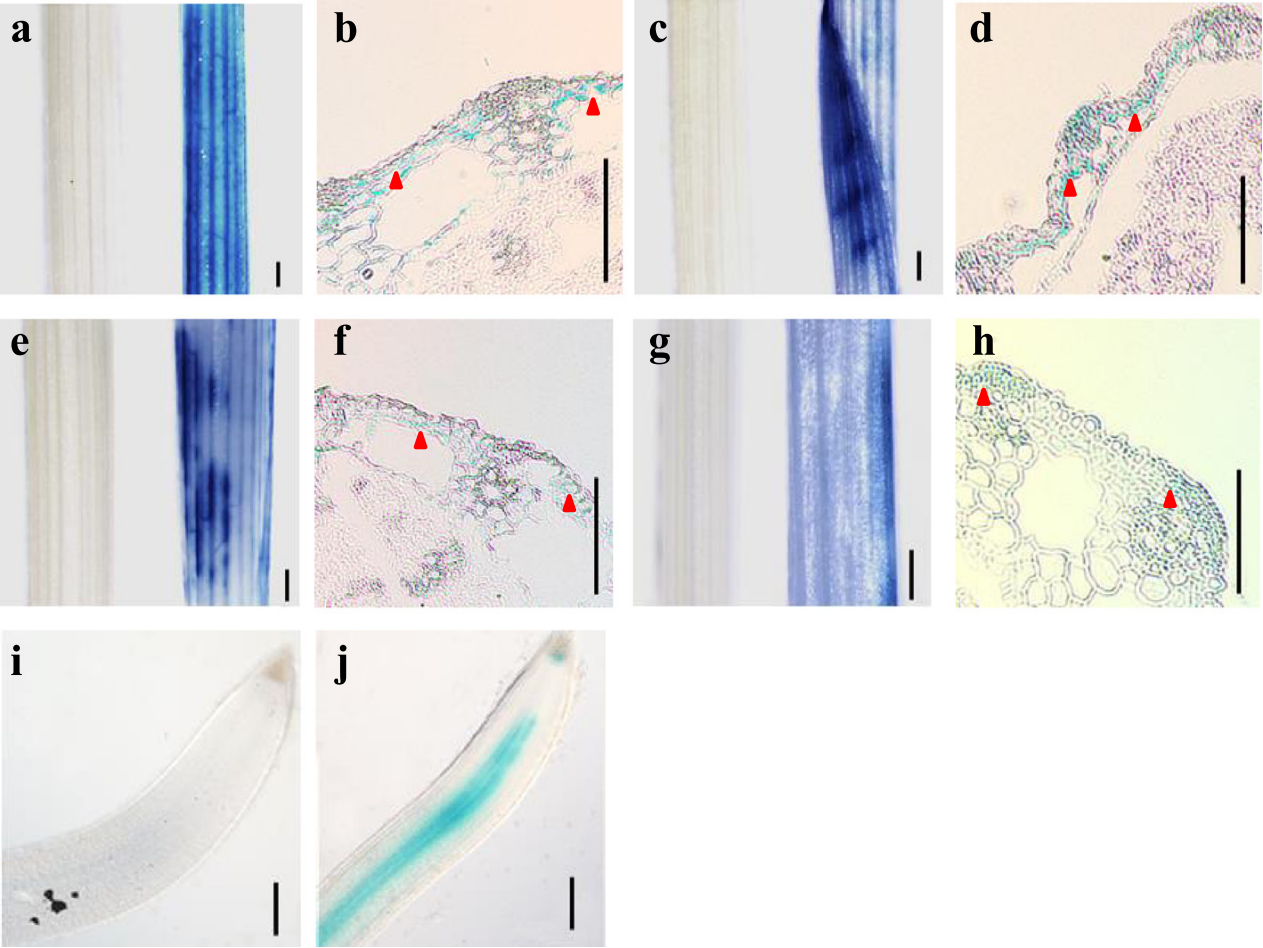
**Patterns of** ***GUS*** **expression in 5 DAG seedlings. (A-H)** Preferential expression of genes in leaf sheaths from Lines 3A-60813 **(a and b)**, 3A-51694 **(c and d)**, 4A-02639 **(e and f)**, and 4A-01614 **(g and h). (a, c, e, and g)** Seedlings were grown under N-sufficient conditions (left) or N-deficient conditions (right). **(b, d, f, and h)** Cross section of leaf sheath under N-starvation. **(i and j)** Preferential expression in vascular cylinders of roots from Line 1B-11001. **(i)** N-sufficient conditions. **(j)** N-deficient conditions. Bar = 200 μm.

We used GUS assays of T-DNA gene trap lines to confirm the N-starvation-responsive TF genes. Those tagging lines were previously generated to have a translational fusion between the tagged gene and *GUS* [31]. Five *GUS*-positive lines displayed N-responsive GUS activity. Although this activity was weak when plants were grown in a standard N-sufficient medium, it was rapidly induced by N-starvation. Under low-N conditions, four lines (3A-60813, 3A-51694, 4A-02639, and 4A-01614) showed preferential GUS-staining in the sheaths (vascular bundles) while one (1B-11001) showed staining in the roots (vascular cylinder) (Figure 4, Table 5). In all five lines, GUS activity was higher for plants in the low-N medium than in the normal MS medium. The Os01g14440 and Os11g02480 genes encode a WRKY TF, Os12g07640 encodes a MYB TF, Os03g55220 encodes a bHelix-loop-helix TF, and Os02g43300 encodes the trihelix TF GTL1. Although the T-DNA vectors carry an intron with triple splicing donors/acceptors at the right border, only one pair of donors and acceptors is utilized that reduces the frequency of translational fusion between the tagged gene and *GUS* [32]. Nonetheless, the *GUS*-trapped TF lines are valuable for investigating their roles during N-starvation.

**Table 5:** **Confirmation of RNA-Seq expression patterns by GUS assays**

**Line no.**	**Locus ID**	**Putative function**	**RNA-Seq log**_**2**_ **(N-/N+) (tissue)**
3A-60813	01 g14440	WRKY1, expressed	1.14 (leaf sheath)
3A-51694	11 g02480	WRKY46, expressed	1.30 (leaf sheath)
4A-02639	12 g07640	MYB family transcription factor, putative, expressed	1.54 (leaf sheath)
4A-01614	03 g55220	bHelix-loop-helix transcription factor	1.38 (leaf sheath)
1B-11001	02 g43300	Trihelix transcription factor GTL1	1.13 (root)

### Analysis of alternative splicing

**Figure 5:**
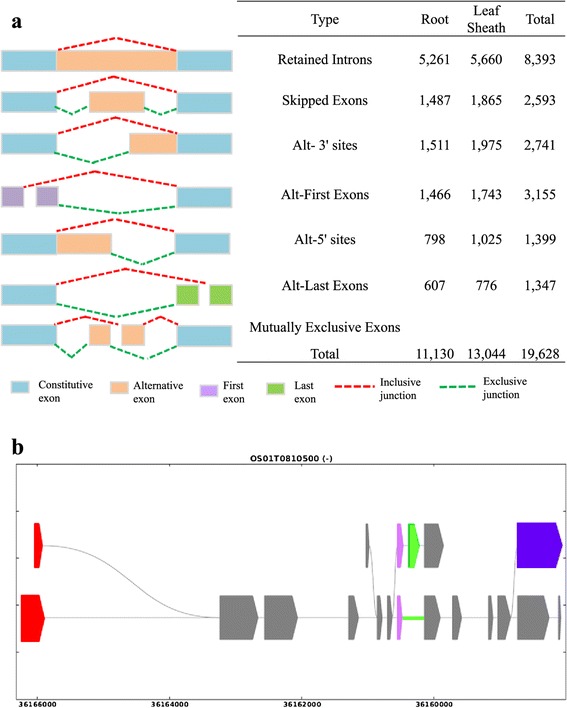
**Analysis of alternative splicing. (a)** Left, 6 types of AS events; right, total numbers of events, including annotated and newly identified splicings. **(b)** Example of NTR (Chromosome 1: 34,427,712-34,436,315). Four types of AS events are indicated. Blue frame, ‘intron retention’; red, ‘alternative first exon’; pink, ‘5′ site’; and green, ‘skipped exon’.

Alternative splicing (AS) is an important regulatory mechanism common in higher eukaryotes that results in a single gene coding for multiple proteins, thereby enhancing biological diversity [33]. Its products are efficiently identified using high-throughput sequencing techniques [34,35]. To investigate potential splicing junctions, we performed computational analyses that revealed 8,509 multi-exonic genes with 19,628 AS events (Figure 5). These events were categorized into six common types. ‘Intron retention’ was the dominant type (42.8%), which is consistent with previous observations from plants [36,37]. By contrast, ‘exon skipping’ is the most prevalent mechanism in humans and yeast [38,39]. Here, ‘alternative 3’ site’, ‘exon skipping’, and ‘alternative first exon’ accounted for 16.1%, 14.0%, and 13.2%, respectively, of all events. Frequencies were relatively low for ‘alternative 5’ site’ (7.1%) and ‘alternative last exon’ (6.9%) (Figure 5a). These data were consistent with other recent reports for plants [36,37,40,41]. An example of the transcript isoforms is shown in Figure 5b.

Alternative splicing can occur because of environmental factors. For example, expression of *Wdreb2* is activated by cold, drought, salt, or exogenous ABA treatment; depending upon the source of the stress, three transcript forms may be produced [42]. However, we found no significant difference in AS between N-sufficient and -deficient conditions, which suggests that it is not involved in the low-N stress response.

### Novel transcribed regions (NTRs) validated by RT-PCR

**Figure 6:**
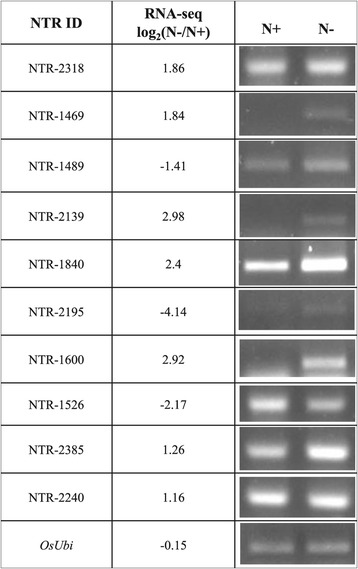
**NTRs validated by RT-PCR.** Semi-quantitative RT-PCR was performed to confirm existence of NTRs preliminarily identified from RNA-Seq analysis. Among 13 NTRs, 10 (77%) were detected in leaf sheath; 7 of those 10 (70%) showed expression patterns consistent with sequencing data.

RNA-Seq technology has revealed novel transcripts that could not be identified previously [43]. Our RNA-Seq data contained 2,986 novel transcribed regions, of which 192 were regulated under the N-deficiency. To confirm their existence, we conducted semi-quantitative reverse transcription PCR with 13 NTRs (Figure 6). Among them, 10 (77%) were detected in the leaf sheath and 7 of those 10 (70%) showed expression patterns consistent with the sequencing data. The three exceptions, with inconsistent patterns, were NTR-1489, NTR-2195, and NTR-2240.

## Conclusion

We performed deep transcriptomic investigations with rice plants and obtained detailed expression profiles for genes involved in responses to low-N stress. These data provide valuable information about the genes (1650 transcripts) induced by N-starvation, expecially the 86 TFs that are key regulators of growth and development. We then confirmed these RNA-Seq data by conducting qRT-PCR and GUS assays of T-DNA tagging lines. In all, 8,509 multi-exonic genes could be linked with 19,628 AS events. However, we found no significant difference in alternative splicing between N-deficient samples and controls. Our data will be useful for identifying N-deficiency-induced genes and investigating the signal transduction pathway of N-utilization.

## Methods

### Plant materials and growth conditions

*Oryza sativa* L. ssp. *japonica* cv. Dongjin rice was used in all experiments. Seeds were surface-sterilized and germinated for two weeks in a Murashige and Skoog medium that lacked a nitrogen source. The seedlings were further grown in an N-sufficient nutrient solution at 28°C/25°C (day/night) under a 14-h photoperiod and 50 to 55% relative humidity. This hydroponic solution, refreshed every 3 d, contained 1.44 mM NH_4_NO_3_, 0.3 mM NaH_2_PO_4_, 0.5 mM K_2_SO_4_, 1.0 mM CaCl_2_, 1.6 mM MgSO_4_, 0.075 μM (NH_4_)_6_Mo_7_O_24_, 18.8 μM H_3_BO_3_, 9.5 μM MnCl_2_, 0.16 μM CuSO_4_, 0.15 μM ZnSO_4_, 35.6 μM FeCl_3_, and 74.4 μM citric acid (pH 5.0) [44]. At the six-leaf stage, the seedlings were divided into two groups: 1) N-starvation, with the amount of NH_4_NO_3_ in the solution reduced to 0.072 mM; and 2) N-sufficient, for which the nutrient solution contained the normal N concentration of 1.44 mM. At 12 h after the treatment began, the total roots and leaf sheaths were harvested from plants in both groups. Each biological replicate constituted a pool of three plants. Two of those replicates were subjected to RNA-sequencing.

### RNA extraction, preparation of cDNA library, and sequencing

Total RNA was prepared using RNAiso Reagent (Takara Bio Inc., Otsu, Japan). Quality was checked with the Agilent 2100 Bioanalyzer (Agilent Technologies, Santa Clara, CA, USA). Total RNA (30 μg) was used for synthesizing complementary DNA (cDNA). After the libraries were constructed, the cDNA was sequenced with the Illumina HiSeq^**TM**^ 2000 according to the manufacturer’s recommendations (http://www.illumina.com).

### Read alignment and assembly

RNA-Seq reads were aligned to the rice reference genomes by the TopHat2 program [30]. That program analyzes the RNA sequences to identify splice junctions between exons by using the ultra-high-throughput short-read aligner Bowtie [29]. Each read was mapped with Cufflinks, which assembled the alignments within the Sequence Alignment/Map file into transfrags [45]. The assembly files were then merged with reference transcriptome annotations into a unified annotation for further analysis [46].

Expression levels for each gene were calculated by quantifying the Illumina reads according to the RPKM method [47]. Replicates were examined independently for statistical analysis. Genes that were differentially expressed by at least two-fold were tested for False Discovery Rate correlations at *p*-values ≤ 0.05 [48]. We also selected any transcripts with RPKM ≥ 2 in at least one cDNA library. Heat maps illustrating patterns for differentially expressed genes were generated as described by Severin *et al.* [49].

### Gene Ontology (GO) term analysis and discovery of alternatively spliced exons

Gene Ontology terms were examined by applying tools for GO enrichment (http://amigo.geneontology.org/cgi-bin/amigo/term_enrichment [50]) and Blast2GO [51], at *p-*values ≤ 0.05. Six basic modes of AS were identified by Cufflinks software, in which differentially spliced exons were detected by comparing pairs of gene models annotated to the same locus [46].

### Identification of novel transcripts

Paired-end reads were mapped to the genome with a spliced-read mapper. Afterward, the reference annotations were used to generate faux-read alignments that covered the transcripts. Those alignments were used together with the spliced-read alignments to produce a reference genome-based assembly. Finally, this assembly was merged with the reference annotations and “noisy” read mappings were filtered, resulting in all reference annotation transcripts in the output as well as novel transcripts [52].

### Real-time RT-PCR

Total RNA was isolated from seedling leaf sheaths and roots, using RNAiso Reagent. For first-strand cDNA synthesis, 1 μg of total RNA was reverse-transcribed in a total volume of 25 μL that contained 10 ng of oligo(dT) 12–18 primer, 2.5 mM dNTPs, and 200 units of AMV Reverse Transcriptase (Promega, Madison, WI, USA) in a reaction buffer. The samples were diluted 10 times prior to PCR. Gene-specific primers were designed using the Oligonucleotide Properties Calculator, or OligoCalc (http://basic.northwestern.edu/biotools/OligoCalc.html). Real-time PCR was performed with 3 μL of template cDNA, 1 μL of forward primer (5 pmol), 1 μL of reverse primer (5 pmol), and 5 μL of SYBR Green mix (Qiagen, Hilden, Germany). Conditions included 5 min of pre-denaturation at 95°C, then 45 cycles of 10 s at 95°C and 20 s at 60°C, followed by steps for dissociation curve generation (15 s at 95°C, 60 s at 60°C, and 15 s at 95°C). To examine the expression of novel transcripts, we performed semi-quantitative RT-PCR with *OsUbiquitin* as the internal reference to equalize the quantity of RNA. After 28 cycles of amplification, PCR products were resolved on a 2% agarose gel and stained with ethidium bromide. All primers are listed in Additional file 7.

### GUS assays

Histochemical GUS-staining was performed according to the method of Jeon *et al.* [53]. Five-d-old seedlings were cut into approximately 1-cm pieces and submerged in a staining solution containing 0.5 M Na_2_HPO_4_ (pH 7.0), 0.5 M NaH_2_PO_4_ (pH 7.0), 0.1% TritonX-100, 0.5 M EDTA (pH 8.0), 1% DMSO, 0.1% X-gluc (5-bromo-4-chloro-3-indolyl-β-d-glucuronic acid/cyclohexylammonium salt), 1 mM K_3_[Fe(CN)_6_], 1 mM K_4_[Fe(CN)_6_], and 5% methanol. The samples were then incubated at 37°C for 12 h. Afterward, the staining solution was replaced with 70% (w/v) ethanol at 65°C to remove the chlorophyll.

### Availability of supporting data

Illumina sequence data are available from NCBI under Short Read Archive accession SRP045923.

## Additional files

Additional file 1: Table S1. The 500 most highly expressed transcripts in leaf sheaths under N-starvation. Additional file 2: Table S2. The 500 most highly expressed transcripts in roots under N-starvation. Additional file 3: Table S3. Leaf sheath transcripts differentially expressed between N-deficient and -sufficient conditions. Additional file 4: Table S4. Root transcripts differentially expressed between N-deficient and -sufficient conditions. Additional file 5: Figure S1. Hierarchical cluster analysis of differentially expressed transcripts due to N-deficiency. Heat map illustrates profiles of 1,650 transcripts differentially expressed due to N-starvation. Red, high expression; blue, low expression. Values on color scale (0 to 10) represent log_2_ (RPKM + 1) for each gene. Additional file 6: Table S5. Transcription factors differentially expressed due to N-starvation. Additional file 7: Table S6. Primer sequences for RT-PCR analyses.

## Abbreviations

AS: Alternative splicing
cDNA: Complementary DNA
GO: Gene ontology
N: Nitrogen
NR: Nitrate reductase
NRT: Nitrate transporter
NTR: Novel transcribed region
PCR: Polymerase chain reaction
qRT-PCR: Quantitative reverse transcription polymerase chain reaction
RNA-Seq: RNA sequencing
RPKM: Reads per kilobase per million reads
TF: Transcription factor

## References

[1] Socolow RH (1999). Nitrogen management and the future of food: lessons from the management of energy and carbon. Proc Natl Acad Sci.

[2] Bi YM, Zhang Y, Signorelli T, Zhao R, Zhu T, Rothstein S (2005). Genetic analysis of *Arabidopsis* GATA transcription factor gene family reveals a nitrate-inducible member important for chlorophyll synthesis and glucose sensitivity. Plant J.

[3] Zhang H (1998). An *Arabidopsis* MADS box gene that controls nutrient-induced changes in root architecture. Science.

[4] Imamura S, Kanesaki Y, Ohnuma M, Inouye T, Sekine Y, Fujiwara T (2009). R2R3-type MYB transcription factor, CmMYB1, is a central nitrogen assimilation regulator in *Cyanidioschyzon merolae*. Proc Natl Acad Sci.

[5] Peng M, Hudson D, Schofield A, Tsao R, Yang R, Gu H (2008). Adaptation of *Arabidopsis* to nitrogen limitation involves induction of anthocyanin synthesis which is controlled by the *NLA* gene. J Exp Bot.

[6] Watanabe CK, Hachiya T, Takahara K, Kawai-Yamada M, Uchimiya H, Uesono Y (2010). Effects of AOX1a deficiency on plant growth, gene expression of respiratory components and metabolic profile under low-nitrogen stress in *Arabidopsis thaliana*. Plant Cell Physiol.

[7] Remans T, Nacry P, Pervent M, Girin T, Tillard P, Lepetit M (2006). A central role for the nitrate transporter NRT2.1 in the integrated morphological and physiological responses of the root system to nitrogen limitation in *Arabidopsis*. Plant Physiol.

[8] Engineer CB, Kranz RG (2007). Reciprocal leaf and root expression of AtAmt1.1 and root architectural changes in response to nitrogen starvation. Plant Physiol.

[9] Yanagisawa S, Akiyama A, Kisaka H, Uchimiya H, Miwa T (2004). Metabolic engineering with Dof1 transcription factor in plants: improved nitrogen assimilation and growth under low-nitrogen conditions. Proc Natl Acad Sci.

[10] Fuentes SI, Allen DJ, Ortiz-Lopez A, Hernandez G (2001). Over-expression of cytosolic glutamine synthetase increases photosynthesis and growth at low nitrogen concentrations. J Exp Bot.

[11] Martin A, Lee J, Kichey T, Gerentes D, Zivy M, Tatout C (2006). Two cytosolic glutamine synthetase isoforms of maize are specifically involved in the control of grain production. Plant Cell.

[12] Yamaya T, Obara M, Nakajima H, Sasaki S, Hayakawa T, Sato T (2002). Genetic manipulation and quantitative-trait loci mapping for nitrogen recycling in rice. J Exp Bot.

[13] Good AG, Johnson SJ, De Pauw M, Carroll RT, Savidov N, Vidmar J (2007). Engineering nitrogen use efficiency with alanine aminotransferase. Can J Bot.

[14] Shrawat AK, Carroll RT, DePauw M, Taylor GJ, Good AG (2008). Genetic engineering of improved nitrogen use efficiency in rice by the tissue-specific expression of alanine aminotransferase. Plant Biotechnol J.

[15] Bi YM, Kant S, Clarke J, Gidda S, Ming F, Xu J (2009). Increased nitrogen-use efficiency in transgenic rice plants over-expressing a nitrogen-responsive early nodulin gene identified from rice expression profiling. Plant Cell Environ.

[16] Wang R, Okamoto M, Xing X, Crawford NM (2003). Microarray analysis of the nitrate response in *Arabidopsis* roots and shoots reveals over 1,000 rapidly responding genes and new linkages to glucose, trehalose-6-phosphate, iron, and sulfate metabolism. Plant Physiol.

[17] Peng M, Bi YM, Zhu T, Rothstein SJ (2007). Genome-wide analysis of *Arabidopsis* responsive transcriptome to nitrogen limitation and its regulation by the ubiquitin ligase gene *NLA*. Plant Mol Biol.

[18] Palenchar PM, Kouranov A, Lejay LV, Coruzzi GM (2004). Genome-wide patterns of carbon and nitrogen regulation of gene expression validate the combined carbon and nitrogen (CN)-signaling hypothesis in plants. Genome Biol.

[19] Bi Y-M, Wang R-L, Zhu T, Rothstein SJ (2007). Global transcription profiling reveals differential responses to chronic nitrogen stress and putative nitrogen regulatory components in *Arabidopsis*. BMC Genom.

[20] Lian X, Wang S, Zhang J, Feng Q, Zhang L, Fan D (2006). Expression profiles of 10,422 genes at early stage of low nitrogen stress in rice assayed using a cDNA microarray. Plant Mol Biol.

[21] Beatty PH, Shrawat AK, Carroll RT, Zhu T, Good AG (2009). Transcriptome analysis of nitrogen-efficient rice over-expressing alanine aminotransferase. Plant Biotechnol J.

[22] Cai H, Lu Y, Xie W, Zhu T, Lian X (2012). Transcriptome response to nitrogen starvation in rice. J Biosci.

[23] Xu Z, Zhong S, Li X, Li W, Rothstein SJ, Zhang S (2011). Genome-wide identification of microRNAs in response to low nitrate availability in maize leaves and roots. PLoS One.

[24] Humbert S, Subedi S, Cohn J, Zeng B, Bi YM, Chen X (2013). Genome-wide expression profiling of maize in response to individual and combined water and nitrogen stresses. BMC Genom.

[25] Brouillette LC, Donovan LA (2011). Nitrogen stress response of a hybrid species: a gene expression study. Ann Bot.

[26] Suenaga A, Moriya K, Sonoda Y, Ikeda A, von Wirén N, Hayakawa T (2003). Constitutive expression of a novel-type ammonium transporter *OsAMT2* in rice plants. Plant Cell Physiol.

[27] Sakai H, Lee SS, Tanaka T, Numa H, Kim J, Kawahara Y (2013). Rice Annotation Project Database (RAP-DB): an integrative and interactive database for rice genomics. Plant Cell Physiol.

[28] Kawahara Y, de la Bastide M, Hamilton JP, Kanamori H, McCombie WR, Ouyang S (2013). Improvement of the *Oryza sativa* Nipponbare reference genome using next generation sequence and optical map data. Rice.

[29] Langmead B, Trapnell C, Pop M, Salzberg SL (2009). Ultrafast and memory-efficient alignment of short DNA sequences to the human genome. Genome Biol.

[30] Kim D, Pertea G, Trapnell C, Pimentel H, Kelley R, Salzberg SL (2013). TopHat2: accurate alignment of transcriptomes in the presence of insertions, deletions and gene fusions. Genome Biol.

[31] Jeong DH, An S, Kang HG, Moon S, Han JJ, Park S (2002). T-DNA insertional mutagenesis for activation tagging in rice. Plant Physiol.

[32] Kim SL, Choi M, Jung KH, An G (2013). Analysis of the early-flowering mechanisms and generation of T-DNA tagging lines in Kitaake, a model rice cultivar. J Exp Bot.

[33] Black DL (2003). Mechanisms of alternative pre-messenger RNA splicing. Annu Rev Biochem.

[34] David CJ, Manley JL (2008). The search for alternative splicing regulators: new approaches offer a path to a splicing code. Genes Dev.

[35] Matlin AJ, Clark F, Smith CW (2005). Understanding alternative sp licing: towards a cellular code. Nat Rev Mol Cell Biol.

[36] Ner-Gaon H, Halachmi R, Savaldi-Goldstein S, Rubin E, Ophir R, Fluhr R (2004). Intron retention is a major phenomenon in alternative splicing in *Arabidopsis*. Plant J.

[37] Wang BB, Brendel V (2006). Genomewide comparative analysis of alternative splicing in plants. Proc Natl Acad Sci.

[38] Sultan M, Schulz MH, Richard H, Magen A, Klingenhoff A, Scherf M (2008). A global view of gene activity and alternative splicing by deep sequencing of the human transcriptome. Science.

[39] Wang ET, Sandberg R, Luo S, Khrebtukova I, Zhang L, Mayr C (2008). Alternative isoform regulation in human tissue transcriptomes. Nature.

[40] Marquez Y, Brown JW, Simpson C, Barta A, Kalyna M (2012). Transcriptome survey reveals increased complexity of the alternative splicing landscape in *Arabidopsis*. Genome Res.

[41] Zhang G, Guo G, Hu X, Zhang Y, Li Q, Li R (2010). Deep RNA sequencing at single base-pair resolution reveals high complexity of the rice transcriptome. Genome Res.

[42] Egawa C, Kobayashi F, Ishibashi M, Nakamura T, Nakamura C, Takumi S (2006). Differential regulation of transcript accumulation and alternative splicing of a *DREB2* homolog under abiotic stress conditions in common wheat. Genes Genet Syst.

[43] Wang Z, Gerstein M, Snyder M (2009). RNA-Seq: a revolutionary tool for transcriptomics. Nat Rev Genet.

[44] Yoshida S, Forno DA, Cock JH, Gomez KA (1976). Laboratory manual for physiological studies of rice. The International Rice Research Institute.

[45] Trapnell C, Williams BA, Pertea G, Mortazavi A, Kwan G, van Baren MJ (2010). Transcript assembly and quantification by RNA-Seq reveals unannotated transcripts and isoform switching during cell differentiation. Nat Biotechnol.

[46] Trapnell C, Roberts A, Goff L, Pertea G, Kim D, Kelley DR (2012). Differential gene and transcript expression analysis of RNA-seq experiments with TopHat and Cufflinks. Nat Protoc.

[47] Mortazavi A, Williams BA, McCue K, Schaeffer L, Wold B (2008). Mapping and quantifying mammalian transcriptomes by RNA-Seq. Nat Meth.

[48] Anders S, Huber W. Differential expression of RNA-Seq data at the gene level-the DESeq package. Eur Mol Biol Lab. 2013.

[49] Severin AJ, Woody JL, Bolon YT, Joseph B, Diers BW, Farmer AD (2010). RNA-Seq atlas of *Glycine max*: a guide to the soybean transcriptome. BMC Plant Biol.

[50] Carbon S, Ireland A, Mungall CJ, Shu S, Marshall B, Lewis S (2009). AmiGO: online access to ontology and annotation data. Bioinformatics.

[51] Conesa A, Gotz S (2008). Blast2GO: a comprehensive suite for functional analysis in plant genomics. Intl J Plant Genom.

[52] Roberts A, Pimentel H, Trapnell C, Pachter L (2011). Identification of novel transcripts in annotated genomes using RNA-Seq. Bioinformatics.

[53] Jeon JS, Lee S, Jung KH, Jun SH, Jeong DH, Lee J (2000). T-DNA insertional mutagenesis for functional genomics in rice. Plant J.

