# Supplementary figures and images for: Transcriptome analysis of nitrogen-starvation-responsive genes in rice
Source: BMC Plant Biol. 2015 Feb 3;15:31. doi: 10.1186/s12870-015-0425-5 (PMC4333837; doi:10.1186/s12870-015-0425-5)

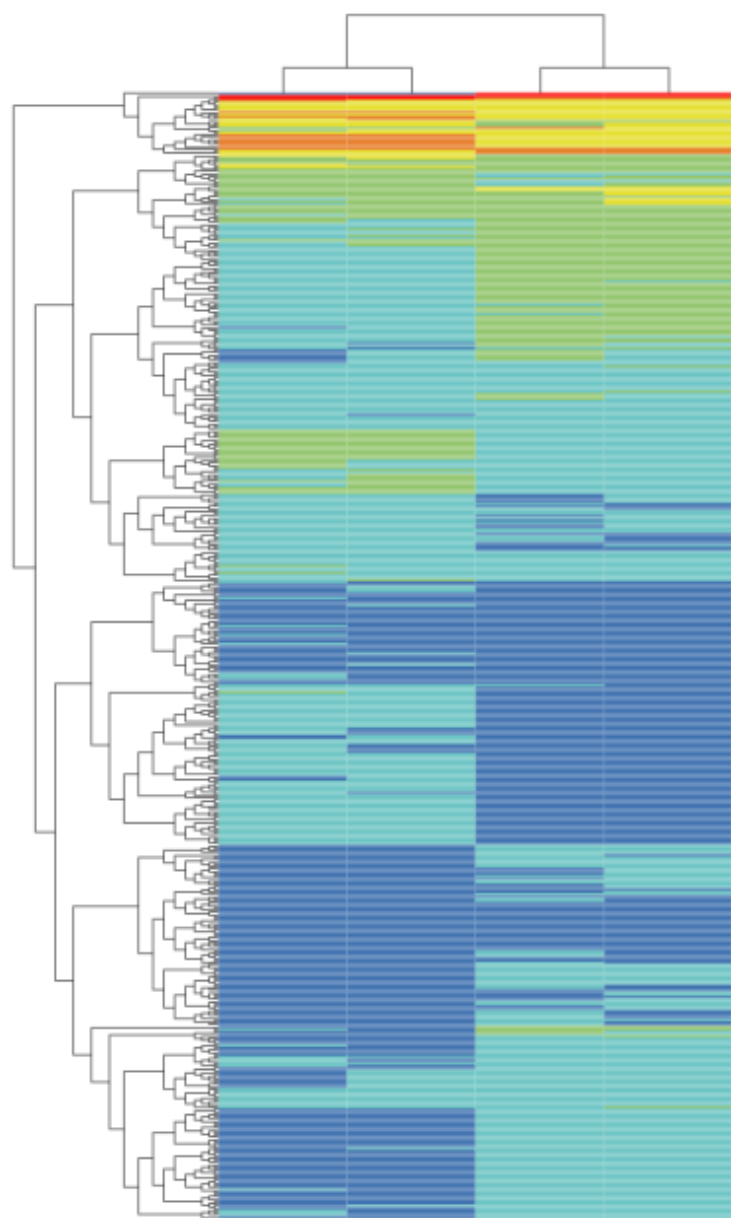

N+      N-

Roots

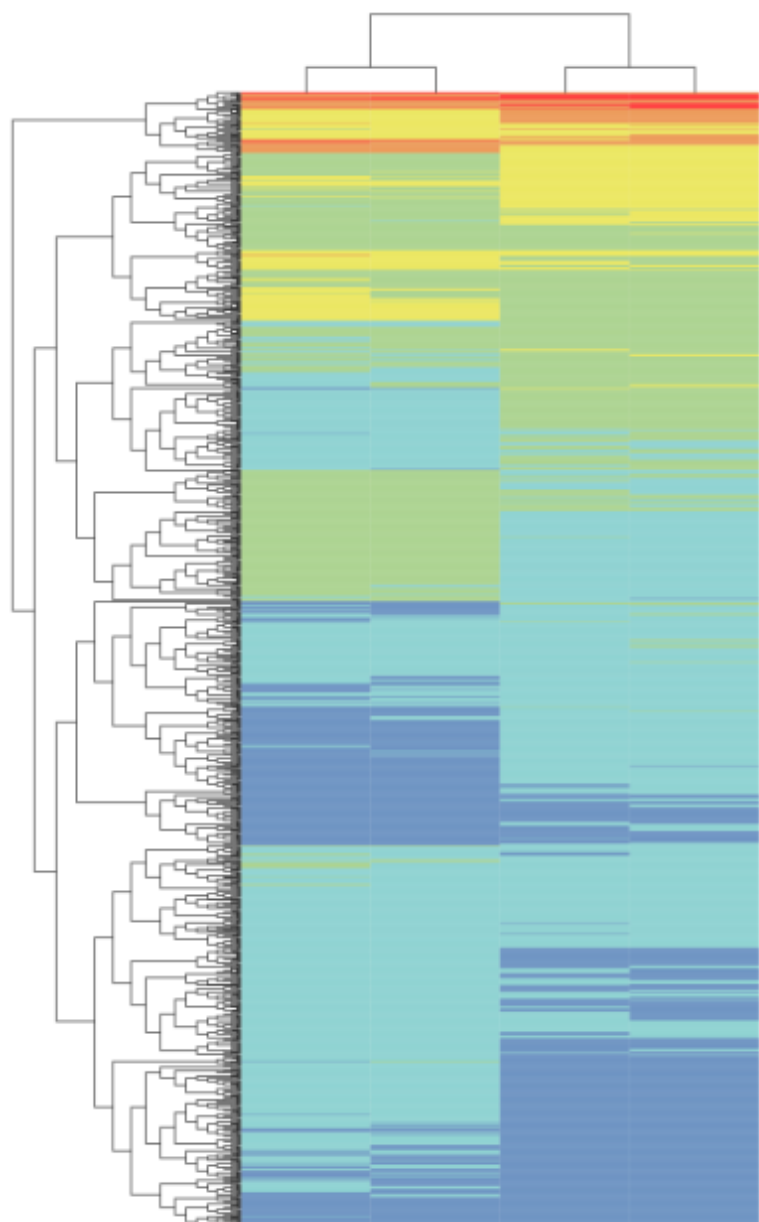

N+      N-

Leaf sheaths

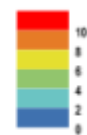

Supplement: Additional file 5: Figure S1. — Hierarchical cluster analysis of differentially expressed transcripts due to N-deficiency. Heat map illustrates profiles of 1,650 transcripts differentially expressed due to N-starvation. Red, high expression; blue, low expression. Values on color scale (0 to 10) represent log2 (RPKM + 1) for each gene. [file 12870_2015_425_MOESM5_ESM.pdf]
